# Monthly Report of Diseases

**Published:** 1800-12

**Authors:** 


### MONTHLY REPORT of DISEASES,

### Admitted under the Care of the PHYSICIANS of the FINSBURY DISPENSARY, St. John's Square, Clerkenwell.

From October 20 to November 20, 1800.

| | | | |
|---|---|---|---|
| Continued Fever - - - | 58 | Gaftrodynia and Enterodynia | 6 |
| Intermittent Fever - - | 1 | Menorrhagia - - - - - | 2 |
| Pneumonia - - - - - - | 8 | Chlorofis and Amenorrhœa | 9 |
| Hæmoptyfis - - - - - | 2 | Hyfteria - - - - - - | 2 |
| Phthifis Pulmonalis - - | 8 | Epilepfy - - - - - - | 3 |
| Catarrh - - - - - - | 16 | Dropfy - - - - - - - | 5 |
| Cough and Dyfpnœa - | 48 | Afthenia - - - - - - | 15 |
| Rheumatifm - - - - - | 21 | Palfy - - - - - - - - | 2 |
| Cynanche Parotidea - - | 1 | Cephalæa - - - - - - | 6 |
| Eryfipelas - - - - - - | 1 | Gout - - - - - - - - | 1 |
| Peritonitis - - - - - - | 2 | Aneurifmus Aortæ - - | 2 |
| Nephritis - - - - - - | 1 | Hæmorrhois - - - - - | 3 |
| Diarrhœa - - - - - - | 3 | Colica Pictonum - - - | 1 |
| Dyfentery - - - - - - | 3 | Scorbutus - - - - - - | 1 |
| Hypochondriafis - - - | 2 | Chronic Cutaneous Difeafes | 9 |
| Dyfpepfia - - - - - - | 5 | Difeafes of Infants - - | 16 |

The autumnal epidemics have retired with the feafon, and the winter has been ufhered in by its ufual train of attendant difeafes. Thofe of the lungs, comprized under the terms pneumonia, catarrh, phthifis, cough and dyfpnœa, hæmoptyfis, &c. have become univerfal fince our laft report; and from their importance and the aggravated ftate of their fymptoms, now occupy a principal fhare of medical attention. Thefe complaints, from the peculiar character of the climate, are remarkably common in this ifland; and from the circumftances of a large and populous city, prevail among the inhabitants of London in a much greater proportion than among thofe of the country. The refpiration of more than a million of inhabitants, and of the horfes and other animals fubfervient to their ufe, within fo confined a fpace, conftantly exhaufts a confiderable portion of the vital or oxygenous part of the atmofphere, and imparts to it, at the fame time, an almoft equal quantity of a gafeous gaseous fluid, positively deleterious to animal life. The continual burning of fuel, on a prodigious scale, not only contributes to the same effect, but furnishes an immense mass of smoke and sooty particles, enveloping the city and its environs to a considerable extent. The air is rendered further impure by the effluvia from common sewers, and repositaries of filth and excremental matters; from stables, slaughter-houses, many sorts of offensive manufactories, the refuse of markets, provision-shops, &c. A quantity of dust is likewise put in motion, and diffused abroad, by the continual stirring of the inhabitants, and by the operations of trade, and of domestic cleanliness. The air thus deprived, in some degree, of its salutary property, and impregnated with noxious substances both chemical and mechanical, is generally, at this period of the year, loaded also with a thick and sluggish fog. When the agent immediately subservient to the function of respiration is so contaminated, it is not wonderful that the function itself should, in consequence, be impeded and deranged. Hence an habitual cough is remarkably frequent among the inhabitants of London, laying a foundation in some for the phthisis pulmonalis, and degenerating in others to a constant state of dyspnœa, with increased secretion from the bronchial vessels. This morbid condition of the lungs becomes aggravated throughout the winter season; and on the special application of cold, or other exciting causes, is, according to the age, constitution, &c. of the patient, often converted into one or other of the species of pneumonia, but principally into that which has been denominated peripneumonia notha. For the same reason also, acute pulmonary diseases are much retarded in their cure, or are protracted to a chronic state.

The continued fever steadily preserves its dominion, and with nearly the same character as during the two preceding months; lately, indeed, it has in several persons been accompanied with an inflammation and slight ulceration of the throat. This circumstance was observed in the fever of the last year, about the same period. It is usually connected with the prevalence of idiopathic anginas; but of these there has lately occurred no instance within our notice, if we except the case of cynanche parotidea.

Although the origin of fever among the poor, may, in general, be easily and distinctly traced, yet on some occasions its source is by no means obvious. Three children, from the age of 6 to 12 years, belonging to a mechanic whose apartments were remarkably clean, and in an airy situation, were attacked in the afternoon of the *same* day by the cold paroxysm of fever, which was soon succeeded by a permanent state of heat, quickness of pulse, and other usual symptoms. In two, the disease terminated favourably in little more than a week; but in the third, it ended fatally about the twenty-first day. The parents being minutely questioned as to the circumstances to which these patients had been exposed previously to the attack of fever, were perfectly sure that they had not been near any person ill of the disease; but mentioned that they sometimes used to play and wander in a neighbouring church-yard, (St.

Luke's) and that their curiofity often excited them to hover over, and look into the graves, at the time of, and immediately after, the ceremony of interring the dead, of whom a great number lately buried in that cemetery, have been the victims of contagious fever.

Of the difeafes of infants, that which moft frequently engages our notice, and baffles our efforts for its removal, is the atrophia. The predifpofition to this morbid ftate, confifts in the weak, fcrophulous, and degenerated ftamina imparted to them by their parents; its exciting caufes are a confined and corrupted air, the want of proper nurfing, unfit and deficient nutriment, and perhaps, but too frequently, the unnatural and premature adminiftration of ardent fpirits. An infant after thriving tolerably well for fome time, perhaps begins to wafte; the abdomen gradually grows hard and tumid; the flefh, as it diminifhes in quantity, becomes foft; and the fkin dry, loofe, and flaccid; the features fhrink, and look pale and fqualid. The alvine difcharges are fometimes too flow, fometimes too frequent, and feldom natural in odour or appearance: the infant is extremely reftlefs and fretful, and has a burning hectic fever, efpecially in the night; a harraffing cough often attends, with much oppreffion of the breathing; the appetite is, for the moft part, keen to the laft. The immediate caufe of this difeafe, is generally an enlargement of the mefenteric glands, and of other parts within the abdomen; fometimes alfo there is a tubercular ftate of the lungs. In the earlier period of this malady, fome little good may be derived from medicinal means, particularly from the judicious ufe of calomel, rhubarb, &c. but unlefs pure air, and proper management at home, co-operate with our endeavours, it is but too common that they prove ultimately ineffectual.

If the late general deficiency of the effential articles of nourifhment has rendered the office of a phyfician, who is employed to a great extent amongft the lower claffes of the community, not only unfpeakably painful to himfelf, but in too large a proportion of cafes almoft entirely unprofitable to his patients, how ufelefs is it to adminifter phyfic to perfons who are wanting food! Medicine is but a poor fubftitute for meat; fo far indeed is the former from performing the office of the latter, that it often even aggravates the fuffering that arifes from the want of it, by awakening an artificial appetite, the violence of which there are no natural means of fubduing.

